# Nalmefene vs. dexmedetomidine for prevention of postoperative hyperalgesia in patients undergoing laparoscopic gynecological surgery with remifentanil infusion: A randomized double-blind controlled trial

**DOI:** 10.3389/fphar.2023.1131812

**Published:** 2023-01-25

**Authors:** Zhen Jia, Yi Chen, Tianyu Gao, Yuan Yuan, Yuxin Zheng, Yegong Xie, Guolin Wang, Yonghao Yu, Linlin Zhang

**Affiliations:** ^1^ Department of Anesthesiology, Tianjin Medical University General Hospital, Tianjin, China; ^2^ Tianjin Research Institute of Anesthesiology, Tianjin, China

**Keywords:** hyperalgesia, nalmefene, pain threshold, remifentanil, dexmedetomidine

## Abstract

Intraoperative remifentanil infusion may paradoxically induce post-surgical hyperalgesia. Dexmedetomidine reportedly reduces opioid-induced hyperalgesia. Nalmefene selectively reverses several side-effects of opioids without impairing analgesia. Herein, this randomized, double-blind controlled trial investigated whether nalmefene, dexmedetomidine, and both drugs combined prevent remifentanil-induced hyperalgesia. One hundred and fifty patients undergoing elective laparoscopic gynecological surgery under desflurane anesthesia randomly received either intraoperative sufentanil 0.20 μg kg^−1^ (Group S), or remifentanil 0.20 μg kg^−1^ min^−1^ (Group R), or remifentanil and pre-anesthesia nalmefene 0.20 μg kg^−1^ (Group N), or remifentanil and pre-anesthesia dexmedetomidine 0.50 μg kg^−1^ (Group D), or remifentanil and the combination of dexmedetomidine 0.25 μg kg^−1^ and nalmefene 0.10 μg kg^−1^ (Group DN). The threshold of postoperative mechanical hyperalgesia (primary outcome) was measured with von Frey filaments. We also recorded pain intensity, analgesic consumptions, hyperalgesic area, and side-effects for 24 h postoperatively. Compared with Group S, remifentanil reduced hyperalgesic threshold on the forearm [mean 89.4 (SD 13.7) vs. 62.2 (10.7) g, *p* < 0.001] at postoperative 24 h. Pain threshold on the forearm at postoperative 24 h was significantly lower in Group R than in Groups N, D and DN [62.2 (10.7) vs. 71.1 (12.3), 72.4 (12.9) and 78.0 (13.8) g]. Compared with Group R, Postoperative pain intensity, analgesic consumption and hyperalgesic area were lower likewise in Groups D and DN. However, the incidence of intraoperative bradycardia was lower and post-anesthesia recovery time was shorter in Group DN than Group D. Preoperative therapy of dexmedetomidine and nalmefene combined attenuates postoperative hyperalgesia in patients undergoing laparoscopic gynecological surgery under desflurane-remifentanil anesthesia.

## Introduction

Remifentanil, as an ultra-short-acting μ-opioid receptor agonist, represents the current mainstay for intraoperative analgesia in clinics (Egan, 1995; Komatsu et al., 2007). Unfortunately, accumulating evidence indicates that intraoperative exposure to remifentanil can elicit a paradoxical state of hypersensitivity to noxious stimuli after surgery, termed remifentanil-induced hyperalgesia (RIH) (Mercieri et al., 2017; Santonocito et al., 2018; Hu et al., 2020). RIH may aggravate postoperative pain and promote pain chronification (Shin et al., 2010; Araldi et al., 2015; de Hoogd et al., 2016; Yu et al., 2016). Therefore, it will be of great importance to find effective approaches for the prevention of RIH in patients.

A highly selective α_2_-adrenergic receptor agonist dexmedetomidine is proposed as an adjunctive therapy for enhancing the analgesia of opioids and reducing the requirement of anesthetics in the perioperative period (Blaudszun et al., 2012; Grape et al., 2019). Experimental data from animals and patients reveal the anti-hyperalgesic effect of dexmedetomidine on RIH (Lee et al., 2013; Yuan et al., 2017; Wu et al., 2021). However, dexmedetomidine has potential side-effects and should be systemically administered with discretion in patients with hypotension and/or bradycardia (Ihmsen and Saari, 2012).

Nalmefene and naloxone, as μ-opioid receptor antagonists, compete with opioids for µ2 receptor binding and precipitate opioids-µ1 receptor binding, thereby reducing opioids-related adverse effects including respiratory depression, nausea, vomiting and pruritus without compromising antinociception (Rawal et al., 1986; Joshi et al., 1999; Crain and Shen, 2000; Kyhl et al., 2016). Compared with naloxone, nalmefene has rapid onset, high potency, long duration and low side-effects (Glass et al., 1994; Kim et al., 1997). Recent study reports that naloxone at low-dose alleviates RIH in rodents and patients (Aguado et al., 2013; Koo et al., 2017). However, nalmefene and nalmefene combined with dexmedetomidine have never been investigated for RIH-relief in the clinical setting.

This randomized, double-blinded, placebo-controlled study was designed to compare nalmefene and dexmedetomidine separately and in combination to reduce postoperative hyperalgesia in patients undergoing laparoscopic gynecologic surgery under remifentanil-desflurane anesthesia. Our findings may offer a possibility for a novel recommendation for prevention of remifentanil-induced hyperalgesia.

## Materials and methods

### Study design

This study was approved by the Tianjin Medical University General Hospital Ethic Committee (Tianjin, China; Approval Number: IRB 2017-009-01), and the study protocol was registered (www.clinicaltrials.gov; Identifier: NCT03096730), and written informed consent was obtained from all patients.

Patients were randomly allocated to one of five groups: Patients in Group S receiving intraoperative sufentanil 0.30 μg kg^−1^ and pre-anesthesia placebo (normal saline, similar volume of nalmefene); patients in Group R receiving intraoperative remifentanil 0.30 μg kg^−1^ min^−1^ and pre-anesthesia placebo; patients in Group N receiving intraoperative remifentanil 0.30 μg kg^−1^ min^−1^ and pre-anesthesia nalmefene (Tiantaishan Medicine Co., Chengdu, China) 0.20 μg kg^−1^; patients in Group D receiving intraoperative remifentanil 0.30 μg kg^−1^ min^−1^ and pre-anesthesia dexmedetomidine (Enhua Medicine Co., Jiangsu, China) 0.50 μg kg^−1^; patients in Group DN receiving intraoperative remifentanil 0.30 μg kg^−1^ min^−1^ and pre-anesthesia nalmefene 0.10 μg kg^−1^ combined with dexmedetomidine 0.25 μg kg^−1^. Nalmefene, dexmedetomidine and placebo were slowly given for 10 min before the induction of anesthesia.

All patient assignments were guided by a computer-generated random number system and individually sealed envelope. Study medication were provided by the hospital pharmacy and administered by an anesthesiologist not involved in the intraoperative management and data collection. Patients were blinded to the group allocation. Primary and secondary outcomes were assessed by another anesthesiologist responsible for the data collection, but not directly involved in the treatment of the patients and who was blinded to randomization.

### Study inclusion and exclusion criteria

Patients undergoing elective laparoscopic gynecological surgery were screened and enrolled between 4 April 2017 and 20 December 2017. Inclusion criteria were patients aged 20–64 years; American Society of Anesthesiologists physical status I or II; cognitive capacity to use the patient-controlled analgesia (PCA). The exclusion criteria were as follows: bronchial asthma; coronary heart disease; severe hypertension; diabetes mellitus; obesity (BMI >30 kg/m^2^); cardiac, hepatic, and renal dysfunction; psychiatric disease; history of chronic pain; history of alcohol or opioid abuse; chronic use of opioids; intake of any analgesic within 48 h before surgery; pregnancy; allergy and contraindication to dexmedetomidine or nalmefene; contraindication for the use of PCA; or incapacity to comprehend pain assessment. After randomization and allocation, patients were withdrawn if laparoscopy was converted to open surgery or if protocol was violation.

### Interventions and anesthesia

All surgical procedures were performed by senior surgeons. Patients fasted preoperatively. Upon arrival at the operating room, the patients were generally monitored by non-invasive blood pressure, ECG, heart rate (HR), pulse oximetry, and bispectral index (BIS). A peripheral intravenous line in the left arm and urinary catheter were attached before induction of anesthesia.

Induction was performed with midazolam 0.05 mg kg^−1^, sufentanil 0.20 μg kg^−1^, and etomidate 0.30 mg kg^−1^, and tracheal intubation was facilitated with rocuronium 0.70 mg kg^−1^. After intubation, all the patients were mechanically ventilated [end-tidal carbon dioxide values of 35–45 mmHg]. Anesthesia was maintained with sufentanil injection or continuous remifentanil (RenFu Co., Hubei, China) infusion as an intraoperative analgesic, and desflurane (Baxter Co., Shanghai, China) as an initial 1.3 minimal alveolar concentration (MAC) and oxygen–air mixture (fraction of oxygen, 50%). The depth of anesthesia was adjusted during surgery by 1% stepwise titration of desflurane, based on targeting BIS (45–60) and hemodynamic changes: HR exceeding pre-induction values by 15% and mean arterial blood pressure (MAP) exceeding baseline values by 20% or <60 mmHg for at least 1 min. Rocuronium (0.30 mg kg^−1^) was administered intermittently i. v. during anesthesia. If bradycardia (HR < 45 beats·min^−1^) and continuous hypotension (MAP <60 mmHg) persisted, additional fluid infusion, atropine (0.50 mg), and phenylephrine (0.10 mg) were also administered. During skin closure, desflurane and remifentanil were stopped, and tropisetron (2 mg) was injected. Residual neuromuscular block was antagonized by neostigmine 0.04 mg kg^−1^ and atropine 0.01 mg kg^−1^ when the tidal volume of spontaneous breathing exceeded 200 ml. When BIS value reached 80, response to oral command was observed, followed by eye opening and spontaneous breathing rate exceeding 10 bpm, the patient was extubated and moved to the postanesthetic care unit (PACU) for recovery at least 1 h.

### Outcomes

RIH was characterized by the significant reduction in pain threshold to the mechanical stimuli on the dominant inner forearm (primary outcome) at 24 h after surgeries and remifentanil infusion as compared to baseline. Hyperalgesia area, pain threshold around the incision, pain intensity, cumulative sufentanyl consumption, and side-effects were the secondary outcomes investigated for 24 h after surgery.

The mechanical pain threshold was assessed using 20 hand-held von Frey filaments (North Coast Medical Inc., Gilroy, CA, United States) in an area 2–5 cm around the incision at 12 predefined positions in all four directions and on the dominant inner forearm according to our previous reports (Zhang et al., 2016). The mechanical hyperalgesia threshold was defined as the smallest force (in grams) necessary to bend a von Frey filament that was detected as painful by the patient (Bornemann-Cimenti et al., 2012; Fechner et al., 2013; Zhang et al., 2016). Also, the normalized area of hyperalgesia around the incision was measured at 24 h after surgery as previously described (Bornemann-Cimenti et al., 2012; Fechner et al., 2013; Zhang et al., 2016).

Pain intensity was evaluated on an 11-point numerical rating scale (NRS): 0 = no pain; and 10 = worst pain imaginable. The NRS score for pain at rest and after movement was assessed at 1, 3, 6, 12, and 24 h after surgery. Movement was specified as active mobilization and weight bearing while escaping any harm (Zhang et al., 2016; Zhang et al., 2017). First postoperative pain (NRS >4) was primarily managed by sufentanil titration, which was administered in 3 µg doses at intervals of 3 min until NRS <4 (Zhang et al., 2016; Zhang et al., 2017). However, sufentanil titration was discontinued if the Ramsay score (1 = anxious and agitated or restless, or both; 2 = cooperative, oriented, and tranquil; 3 = responds to command only; 4 = asleep, but has a brisk response to light tactile stimulus or a simple verbal command; 5 = asleep, but arousable only by strong physical stimulus; and 6 = asleep, unarousable) was >3, peripheral oxygen saturation decreased <92%, or breathing rate was <10 bpm. The time and total dose of first postoperative sufentanil were documented in the PACU. Furthermore, each patient was administered analgesics using a PCA pump containing sufentanil (100 µg) diluted by normal saline to a total volume of 100 ml after discharge from the PACU. The device was set to deliver a basal infusion of 2 ml h^−1^ and bolus doses of .5 ml with a 15 min lockout period. Sufentanil comsumption was recorded at 1, 3, 6, 12, and 24 h after surgery. The incidence of postoperative side-effects was monitored during the 24 h after surgery.

### Statistical analyses

A pilot study was conducted and a power analysis was implemented to calculate the sample size. The mean mechanical pain threshold of the dominant inner forearm before surgery was 96.0 g, whereas the means of the five treatment groups (Group S, Group R, Group N, Group D, and Group DN) at 24 h after surgery were 85.2, 61.5, 67.2, 73.0, and 74.0 g, respectively. We determined a difference of at least 30% (error standard deviation = 26.0) among groups. An *a priori* algorithm was used to estimate the required sample size for analysis of variance (ANOVA) with repeated measures. A sample size of 27 patients per group was found to be sufficient to detect a significant difference (*α* = 5%) with a statistical power (β-value) of 0.8. Presuming a 10% failure rate, we considered increasing the sample size to 30 patients per group.

The Shapiro–Wilk test was used to determine the normality of distribution of the data, and parametric statistics were applied. Homogeneity of variance was verified by the Levene test. Data from the NRS scores, and mechanical pain threshold were analyzed by two-way repeated measures with Bonferroni *post hoc* comparisons. Data from time and total dose of first postoperative sufentanil titration, sufentanil consumption by PCA, and normalized area were analyzed by one-way with Bonferroni *post hoc* comparisons. Other quantitative data, such as age, weight, duration of remifentanil infusion, mean concentration of desflurane, recovery time and extubation time were also analyzed using one-way with Bonferroni *post hoc* comparisons. Simultaneously, the χ^2^ test and Fisher’s exact test were used to analyze categorical variables, such as atropine administration, hypotension, dizziness, nausea, and vomiting. Data were expressed as the mean (SD) or the number of patients/percentage. A *p*-value <0.05 was considered statistically significant. SPSS 21.0 software (SPSS, Inc., Chicago, IL, Unitred States) was used for all statistical analysis.

## Results

### Patient characteristics

**FIGURE 1:**
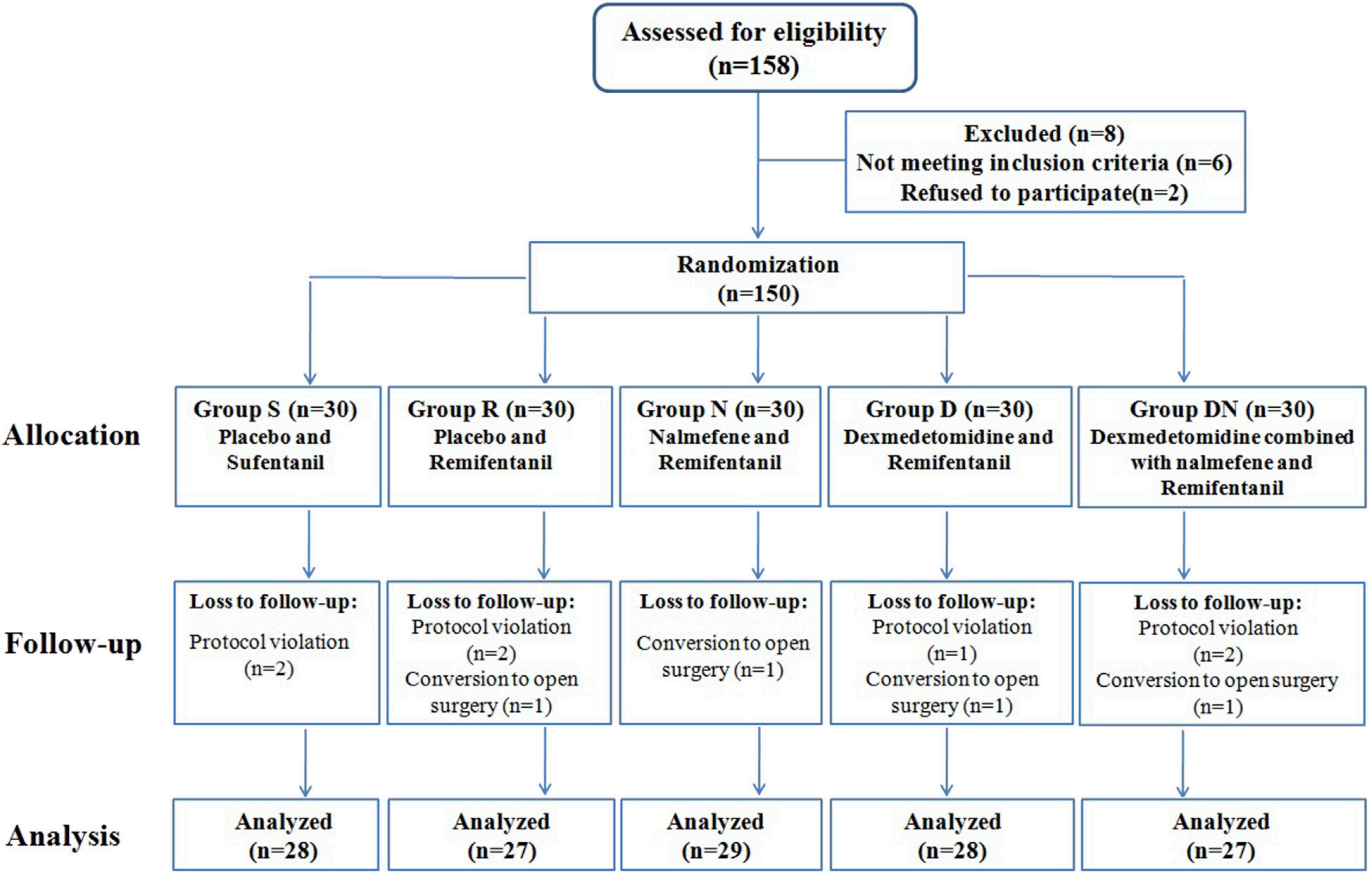
Consolidated Standards of Reporting Trials (CONSORT) flow diagram.

Among the one hundred and fifty-eight patients recruited, one hundred and fifty patients were eligible for inclusion. Eleven patients were withdrawn after protocol violation or conversion to open surgery, and one hundred and thirty-nine patients completed the study (Figure 1). The five groups were balanced in terms of patient characteristics (Table 1).

**TABLE 1:** Patient characteristics and anesthetic data. Group S: Intraoperative sufentanil 0.20 μg kg^−1^ and pre-anesthesia saline; Group R: Intraoperative remifentanil 0.20 μg kg^−1^ min^−1^ and pre-anesthesia saline; Group N: Intraoperative remifentanil 0.20 μg kg^−1^ min^−1^ and pre-anesthesia nalmefene 0.20 μg kg^−1^; Group D: Intraoperative remifentanil 0.20 μg kg^−1^ min^−1^ and pre-anesthesia dexmedetomidine 0.50 μg kg^−1^; Group DN: Intraoperative remifentanil 0.20 μg kg^−1^ min^−1^ and pre-anesthesia nalmefene 0.10 μg kg^−1^ combined with dexmedetomidine 0.25 μg kg^−1^. All patients underwent laparoscopic gynecological surgery under desflurane anesthesia. Values are presented as the mean (SD), or the number of patients/%. *p < 0.05 vs. group R, **p < 0.01 vs. group R. ASA: American Society of Anesthesiologists.

Characteristic	Group S (n = 28)	Group R (n = 27)	Group N (n = 29)	Group D (n = 28)	Group DN (n = 27)
Age (yr)	48(6)	47(7)	47(10)	45(12)	46(9)
Weight (kg)	61(8)	60(7)	58(5)	59(7)	60(8)
ASA status (I)	17/61	19/70	18/62	20/71	19/70
Duration of surgery (min)	92(18)	93(13)	95(21)	94(22)	93(17)
Duration of remifentanil infusion (min)		98(20)	98(19)	97(18)	98(16)
Mean concentration of desflurane (%)	4.2(0.5)	4.4(0.4)	4.5(0.3)	4.1(0.5)	4.3(0.4)
Intraoperative administration					
Phenylephrine	3/11	2/7	1/3	3/11	2/7
Atropine	2/7	2/7	1/3	8/29^*^	3/11

### Intraoperative and post-anesthesia clinical variables in anesthesia

**FIGURE 2:**
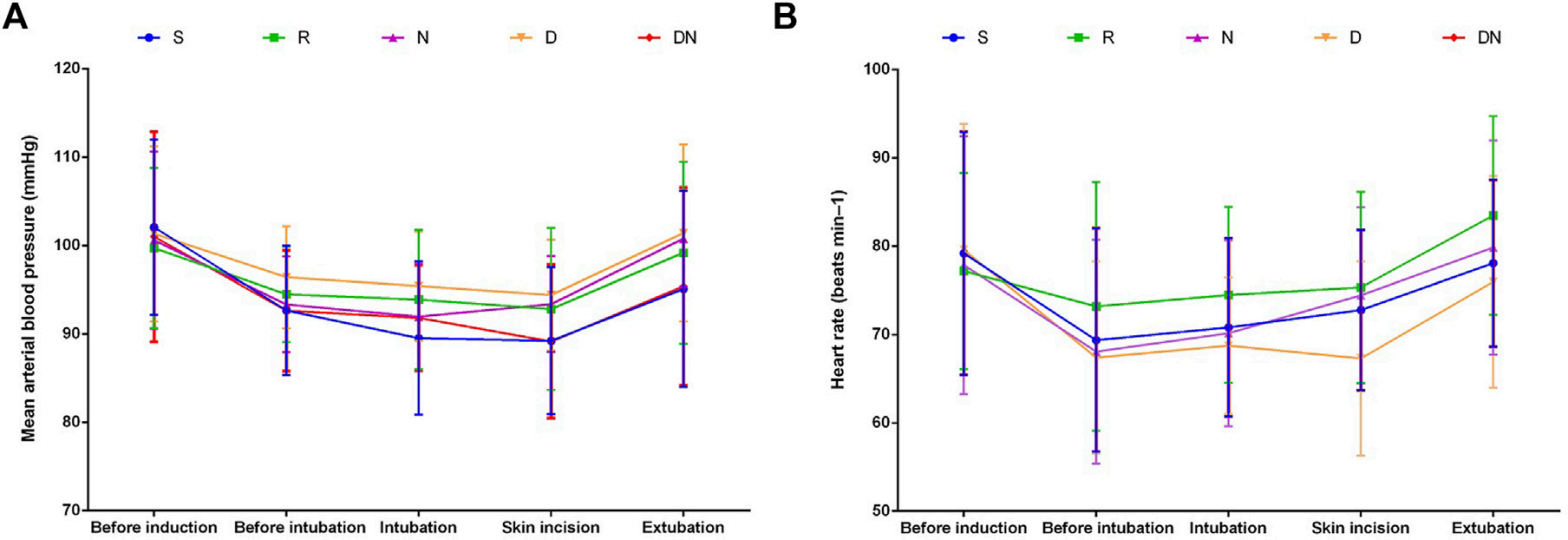
Intraoperative mean arterial blood pressure **(A)** and heart rate **(B)**. Group S: Intraoperative sufentanil 0.20 μg kg^−1^ and pre-anesthesia saline; Group R: Intraoperative remifentanil 0.20 μg kg^−1^ min^−1^ and pre-anesthesia saline; Group N: Intraoperative remifentanil 0.20 μg kg^−1^ min^−1^ and pre-anesthesia nalmefene 0.20 μg kg^−1^; Group D: Intraoperative remifentanil 0.20 μg kg^−1^ min^−1^ and pre-anesthesia dexmedetomidine 0.50 μg kg^−1^; Group DN: Intraoperative remifentanil 0.20 μg kg^−1^ min^−1^ and pre-anesthesia nalmefene 0.10 μg kg^−1^ combined with dexmedetomidine 0.25 μg kg^−1^. All patients underwent laparoscopic gynecological surgery under desflurane anesthesia. Values are presented as mean (SD).

No significant difference was detected between groups with respect to duration of surgery, duration of remifentanil infusion, mean volume of desflurane, intraoperative MAP and HR, and the proportion of patients administered phenylephrine (Table 1; Figure 2). However, Group D exhibited the higher proportion of patients administered atropine as compared to Group R (*p* = 0.044, Table 1). When compared with Group R, Group S exhibited delayed eye opening time [7.1 (1.7) vs. 8.7 (2.2) min, *p* = 0.009] and extubation time [8.4 (1.6) vs. 10.2 (2.3) min, *p* = 0.005]. Simultaneously, patients in Group D exhibited delayed extubation time as compared to patients with remifentanil infusion alone [10.0 (1.7) vs. 8.4 (1.6) min, *p* = 0.012]. However, eye opening time and extubation time did not differ between Groups R, N and DN.

### Mechanical hyperalgesia threshold and area

**FIGURE 3:**
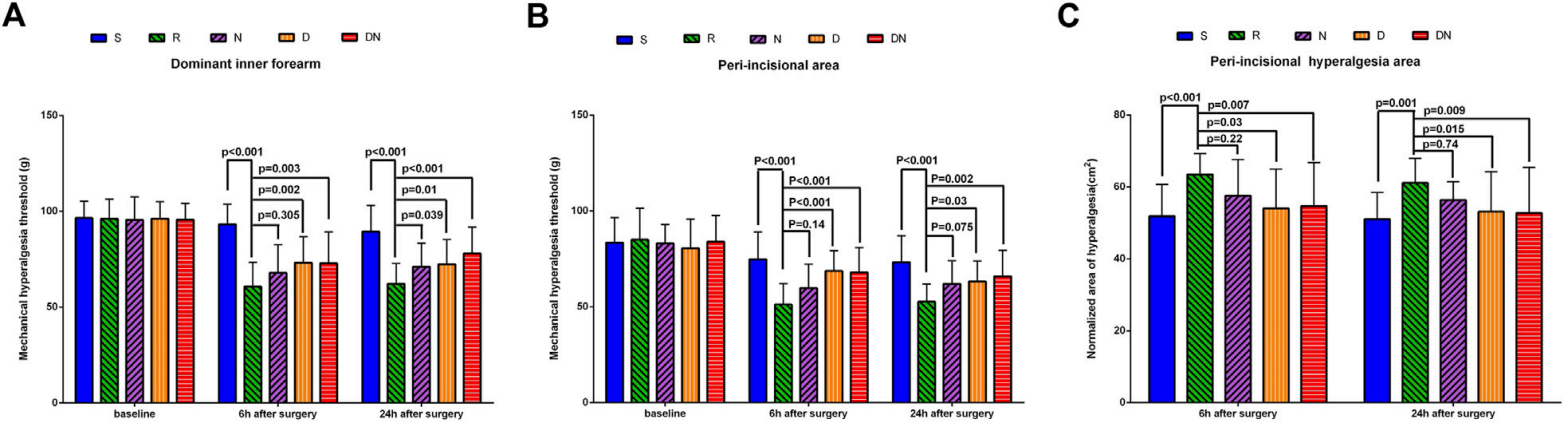
Postoperative mechanical pain threshold and normalized area of hyperalgesia. Mechanical pain threshold on the dominant forearm **(A)** and around the incision **(B)** and normalized area of hyperalgesia **(C)** were tested before and 6 h and 24 h after surgery with Von Frey filaments. Group S: Intraoperative sufentanil 0.20 μg kg^−1^ and pre-anesthesia saline; Group R: Intraoperative remifentanil 0.20 μg kg^−1^ min^−1^ and pre-anesthesia saline; Group N: Intraoperative remifentanil 0.20 μg kg^−1^ min^−1^ and pre-anesthesia nalmefene 0.20 μg kg^−1^; Group D: Intraoperative remifentanil 0.20 μg kg^−1^ min^−1^ and pre-anesthesia dexmedetomidine 0.50 μg kg^−1^; Group DN: Intraoperative remifentanil 0.20 μg kg^−1^ min^−1^ and pre-anesthesia nalmefene 0.10 μg kg^−1^ combined with dexmedetomidine 0.25 μg kg^−1^. All patients underwent laparoscopic gynecological surgery under desflurane anesthesia. Values are presented as mean (SD).

As shown in Figures 3A, B, the baseline mechanical pain threshold on the dominant inner forearm and around the incision was similar among all the groups (*p* > 0.05). Compared with baseline, intraoperative remifentanil infusion induced a robust decrease in pain threshold on the forearm and peri-incisional area at 6 h (*p* < 0.001 and *p* < 0.001) and 24 h (*p* < 0.001 and *p* < 0.001) after surgery. Interestingly, as compared to patients in Group R, higher levels of pain threshold on the forearm and around the incision were detected in Group D at postoperative 6 h (*p* = 0.002 and *p* < 0.001) and 24 h (*p* = 0.01 and *p* = 0.03), and Group DN at postoperative 6 h (*p* = 0.003 and *p* < 0.001) and 24 h (*p* < 0.001 and *p* = .002). Also, patients in Group N showed the higher threshold on the forearm at postoperative 24 h (*p* = 0.039) than that in Group R. However, the threshold around the incision was comparable in Groups R and N at 6 h (*p* = 0.14) and 24 h (*p* = 0.075) after surgery. Besides, similar threshold levels at postoperative 24 h were seen on the forearm (*p* = 0.73) and peri-incisional site (*p* = 0.97) in Groups D and DN.

As shown in Figure 3C, the normalized area of hyperalgesia around the incision in Group R was larger than that in Group S at postoperative 6 h (*p* < 0.001) and 24 h (*p* = 0.001). As compared to Group R, it was downregulated in Groups D and DN at postoperative 6 h (*p* = .03 and *p* = 0.007) and 24 h (*p* = 0.015 and *p* = 0.009). However, there was no significant difference between Group R and Group N at postoperative 6 h (*p* = 0.22) and 24 h (*p* = 0.74).

### Postoperative pain intensity

**FIGURE 4:**
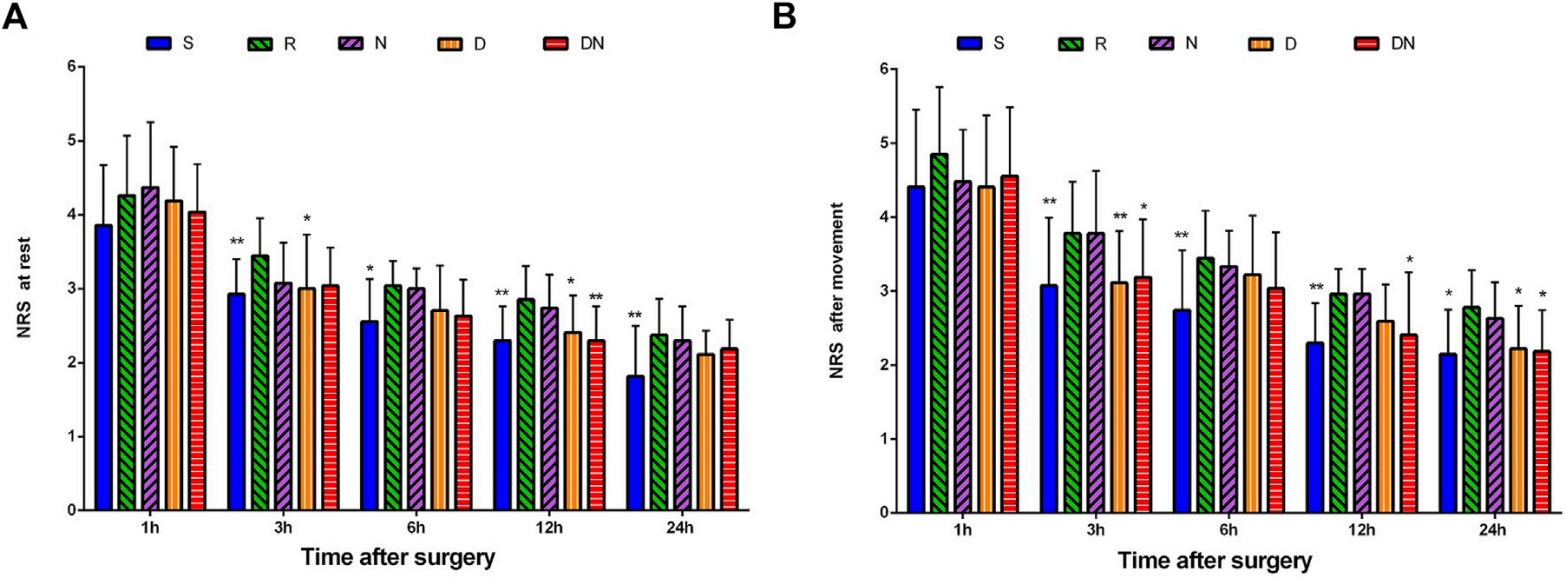
Postoperative pain intensity. The numerical rating scale (NRS) score for pain at rest **(A)** and after movement **(B)** was documented at 1, 3, 6, 12, and 24 h after surgery. Group S: Intraoperative sufentanil .20 μg kg^−1^ and pre-anesthesia saline; Group R: Intraoperative remifentanil 0.20 μg kg^−1^ min^−1^ and pre-anesthesia saline; Group N: Intraoperative remifentanil 0.20 μg kg^−1^ min^−1^ and pre-anesthesia nalmefene 0.20 μg kg^−1^; Group D: Intraoperative remifentanil 0.20 μg kg^−1^ min^−1^ and pre-anesthesia dexmedetomidine 0.50 μg kg^−1^; Group DN: Intraoperative remifentanil 0.20 μg kg^−1^ min^−1^ and pre-anesthesia nalmefene .10 μg kg^−1^ combined with dexmedetomidine 0.25 μg kg^−1^. All patients underwent laparoscopic gynecological surgery under desflurane anesthesia. Values are presented as mean (SD), *p < .05 vs. group R, **p < 0.01 vs. group R.

As shown in Figure 4, NRS scores at rest were higher in Group R than Group S at 3 h (*p* = 0.009), 6 h (*p* = 0.019), 12 h (*p* = 0.004) and 24 h (*p* = 0.004) after surgery. Similarly, patients in Group R showed the higher NRS scores after movement at postoperative 3 h (*p* = 0.003), 6 h (*p* = 0.004), 12 h (*p* = 0.007) and 24 h (*p* = 0.013) as compared to Group S. Strikingly, compared with Group R, Groups D exhibited the lower NRS scores at rest at postoperative 3 h (*p* = 0.042) and 12 h (*p* = 0.039) and NRS scores after movement at 3 h (*p* = 0.007) and 24 h (*p* = 0.046) after surgery. Furthermore, NRS scores at rest were lower in Group DN than Group R at 12 h (*p* = 0.004) after surgery. NRS scores after movement were lower in Group DN than Group R at 3 h (*p* = 0.025), 12 h (*p* = 0.046) and 24 h (*p* = 0.026) after surgery. However, there was no significant difference in NRS scores between Group R and Group N (*p* > 0.05).

### Postoperative sufentanil consumption

As compared to Group S [25.0(7.3) min], patients in Group R [21.4(5.9) min] showed the shorter time to first postoperative sufentanil requirement in the PACU (*p* = 0.001). When compared with Group R, it was prolonged in Group D [24.8(6.3) min, *p* = 0.003] and Group DN [24.4(6.1) min, *p* = 0.013]. However, no significant difference was detected between Group R and Group N [23.7(7.9) min, *p* = 0.133].

Table 2 displays postoperative analgesics administration. As compared to Group R, the demand of sufentanil titration in Group S (*p* < 0.001), Group D (*p* = 0.036) and Group DN (*p* = 0.025) was significantly decreased, whereas there was no significant difference between Group R and Group N (*p* = 0.474). Furthermore, sufentanil consumption by PCA in Group S was significantly less than that in Group R. More importantly, when compared with Group R, sufentanil requirement was greater in Group D during the second 6 h (*p* = 0.007) and the third 6 h (*p* = 0.02) after PCA and in Group DN during the third 6 h (*p* = 0.012) and the fourth 6 h (*p* = 0.036) after PCA. However, no difference was reported between Group R and Group N.

**TABLE 2:** Postoperative sufentanil consumption. Postoperative pain was controlled by sufentanil titration in the PACU, followed by sufentanil infusion *via* PCA. Group S: Intraoperative sufentanil 0.20 μg kg^−1^ and pre-anesthesia saline; Group R: Intraoperative remifentanil 0.20 μg kg^−1^ min^−1^ and pre-anesthesia saline; Group N: Intraoperative remifentanil 0.20 μg kg^−1^ min^−1^ and pre-anesthesia nalmefene 0.20 μg kg^−1^; Group D: Intraoperative remifentanil 0.20 μg kg^−1^ min^−1^ and pre-anesthesia dexmedetomidine 0.50 μg kg^−1^; Group DN: Intraoperative remifentanil 0.20 μg kg^−1^ min^−1^ and pre-anesthesia nalmefene 0.10 μg kg^−1^ combined with dexmedetomidine 0.25 μg kg^−1^. All patients underwent laparoscopic gynecological surgery under desflurane anesthesia. Values are presented as the mean (SD). *p < 0.05 and **p < 0.01 vs. Group R, *p*_1_, Group R vs. Group S; *p*_2_, Group R vs. Group N; *p*_3_, Group R vs. Group D; *p*_4_, Group R vs. Group DN; *p*_5_, Group D vs. Group DN; *p*_0_, comparing groups. PACU, postanesthetic care unit; PCA, patient-controlled analgesia.

Method of sufentanil application	Group S (n = 28)	Group R (n = 27)	Group N (n = 29)	Group D (n = 28)	Group DN (n = 27)	*p*-value					
						*p*_1_	*p*_2_	*p*_3_	*p*_4_	*p*_5_	*p*_0_
By titration in the PACU (μg)											
8.6(3.8)^**^		13.2(2.8)	11.3(3.5)	10.4(3.7)^*^	10.3(4.3)^*^	<0.001	0.474	0.036	0.025	0.99	<0.001
By PCA (μg)											
0–6 h	8.5 (2.3)	10.2 (3.2)	8.7 (2.1)	8.4 (2.5)	8.6 (2.8)	0.21	0.449	0.184	0.273	0.99	0.09
6–12 h	6.4 (2.7)^*^	8.6 (2.8)	7.3 (2.6)	6.1 (2.4) ^**^	6.7 (2.6)	0.033	0.819	0.007	0.11	0.99	0.007
12–18 h	6.3 (2.6)^*^	8.4 (3.3)	6.8 (2.7)	6.2 (2.1)^*^	6.0 (2.2)^*^	0.032	0.251	0.02	0.012	0.99	0.006
18–24 h	4.1 (1.9)^*^	6.1 (2.9)	5.4 (2.6)	4.9 (2.5)	4.1 (2.4) ^*^	0.031	0.99	0.726	0.036	0.99	0.013

### Postoperative side-effects

The incidence of postoperative nausea in Group S were higher than in Group R (*p* = 0.044, Table 3), whereas no differences were found among Groups R, N, D, and DN (*p* > 0.05, Table 3). Additionally, the incidence in postoperative hypotension, bradycardia, vomiting, shivering, somnolence, dizziness and respiratory depression didn’t differ among the five groups (*p* > 0.05, Table 3).

**TABLE 3:** Postoperative side-effects. The incidence of the main adverse effects was evaluated during the first 24 h after surgery. Group S: Intraoperative sufentanil 0.20 μg kg^−1^ and pre-anesthesia saline; Group R: Intraoperative remifentanil 0.20 μg kg^−1^ min^−1^ and pre-anesthesia saline; Group N: Intraoperative remifentanil 0.20 μg kg^−1^ min^−1^ and pre-anesthesia nalmefene 0.20 μg kg^−1^; Group D: Intraoperative remifentanil .20 μg kg^−1^ min^−1^ and pre-anesthesia dexmedetomidine 0.50 μg kg^−1^; Group DN: Intraoperative remifentanil 0.20 μg kg^−1^ min^−1^ and pre-anesthesia nalmefene 0.10 μg kg^−1^ combined with dexmedetomidine 0.25 μg kg^−1^. All patients underwent laparoscopic gynecological surgery under desflurane anesthesia. Values are presented as the number of patients/%. *p*, comparing groups. *p_*1*_ < 0.05 vs. group R.

Side-effect	Group S (*n* = 28)	Group R (*n* = 27)	Group N (*n* = 29)	Group D (*n* = 28)	Group DN (*n* = 27)	*p*-value
Hypotension	3/11	2/7	1/3	5/18	4/15	0.426
Bradycardia	1/4	1/4	2/7	6/21	4/15	0.112
Nausea	8/29^*^	2/7	3/10	4/14	2/7	0.119
Vomiting	2/7	0/0	0/0	1/4	1/4	0.464
Shivering	1/4	3/11	2/7	2/7	2/7	0.882
Somnolence	1/4	3/11	0/0	3/11	2/7	0.376
Dizziness	1/4	2/7	0/0	2/7	0/0	0.375
Respiratory depression	2/7	0/0	0/0	3/11	1/4	0.209

## Discussion

The primary findings of this present study are: First, intraoperative exposure to remifentanil at a clinically relative dose (0.30 μg kg^−1^ min^−1^) induces postoperative hyperalgesia and aggravates postoperative pain in patients after laparoscopic gynecological surgery. Second, pretreatment with nalmefene 0.20 μg kg^−1^ impairs post-surgical RIH but not pain. Third, pretreatment with dexmedetomidine 0.50 μg kg^−1^ effectively alleviates post-surgical RIH and pain but prolongs post-anesthesia recovery and increases the incidence of intraoperative bradycardia. Fourth, preoperative therapy of nalmefene 0.10 μg kg^−1^ and dexmedetomidine 0.25 μg kg^−1^ combined exhibits significant anti-hyperalgesic properties and reduces dexmedetomidine-related side-effects, which may be more beneficial for cardiovascular-compromised patients who would undergo surgery with remifentanil analgesia.

To test hyperalgesic properties of remifentanil in clinical patients, intraoperative sufentanil analgesia was as control. Small incision may just cause mild postoperative pain, and exacerbation of postoperative pain may be primarily associated with RIH development (Shin et al., 2010; Zhang et al., 2016; Colvin et al., 2019). Thus, the laparoscopic gynecologic surgery was chosen. Desflurane was preferred for general anesthesia since it may have less relevance to central pain sensitization (Lee et al., 2013; Yoo et al., 2015). We selected the dose of 0.30 μg kg^−1^ min^−1^ as consecutive remifentanil infusion according to previous reports (Kong et al., 2016; Zhang et al., 2016; Wu et al., 2021). Hyperalgesia was verified by measuring the pain threshold and area to mechanical stimuli using von Frey filaments (Bornemann-Cimenti et al., 2012; Fechner et al., 2013; Zhang et al., 2016; Zhang et al., 2017; Colvin et al., 2019). In order to eliminate the synergic effects of surgery insult on RIH, mechanical pain threshold on the forearm was considered as primary outcome (Bornemann-Cimenti et al., 2012; Zhang et al., 2016). Postoperative pain was evaluated by NRS scores and analgesics consumption after surgery (Koo et al., 2017; Zhang et al., 2017). Not surprisingly, we observed that intraoperative exposure to remifentanil and sufentanil provided similar and sufficient anti-nociception minimizing hemodynamic fluctuations during surgery. However, as compared to sufentanil injection, remifentanil downregulated postoperative mechanical pain threshold both on the forearm and around the incision, and upregulated the normalized area of postoperative hyperalgesia, indicating the existence of RIH in patients undergoing laparoscopic gynecological surgery under desflurane-remifentanil anesthesia. Furthermore, remifentanil increased postoperative pain intensity and analgesics requirements, suggesting the potential role of RIH in surgical nociception stimuli-related pain. Additionally, we detected a time delay of post-anesthesia recovery and a high incidence of nausea after desflurane-sufentanil anesthesia. Taken together, remifentanil occupies a clear advantage in minor operation and is worth popularizing if RIH is effectively controlled.

The activation of N-methyl-d-aspartate (NMDA) receptor is a cardinal feature of central nociception sensitization in the pathogenesis of RIH (Zhang et al., 2014; Shu et al., 2021). Apart from its sedative-analgesic effects, dexmedetomidine possess potent anti-hyperalgesic properties. Specifically, dexmedetomidine administration inhibits the phosphorylation and trafficking of NR2B-containing NMDA receptor in the spinal dorsal horn after remifentanil infusion, attenuating RIH phenotypes in rodents (Zheng et al., 2012; Yuan et al., 2017). Lee and his colleagues found that patients receiving intraoperative dexmedetomidine infusion (an initial dose of 1.0 μg kg^−1^ h^−1^ for 10 min, followed by a continuous infusion of 0.70 μg kg^−1^ h^−1^) efficiently ameliorated RIH symptoms but elevated the frequency of postoperative hypotension and bradycardia (Lee et al., 2013). A recent investigation revealed that a single delivery of dexmedetomidine (30 μg) did not induce significant hypotension and bradycardia in patients undergoing cesarean delivery under spinal or epidural anesthesia, suggesting the safety of dexmedetomidine at a relatively low dose (Lamontagne et al., 2019). Intriguingly, our current results discovered that preoperative bolus of dexmedetomidine at low dose (0.50 μg kg^−1^) attenuated postoperative RIH and pain, as characterized by the abrupt increase in mechanical nociceptive threshold both on the forearm and around the incision, the significant extension in the time of first postsurgical analgesics application, and the dramatic decrease in mechanical hyperalgesic area, pain intensity, and cumulative sufentanil consumption after remifentanil infusion, in agreement with previous reports (Lee et al., 2013; Wu et al., 2021). However, patients with dexmedetomidine (0.50 μg kg^−1^) exhibited delayed extubation, as well as high incidence of intraoperative bradycardia, which is different from the latest study by Wu and his colleagues (Wu et al., 2021). Perhaps, it is because timing administered dexmedetomidine is different. Wu et al. selected the single injection of dexmedetomidine before skin closure (Wu et al., 2021). Anyway, systemic therapy of dexmedetomidine at low dose is an effective approach for preventing RIH after surgery in clinics, but it should still be given cautiously in patients with bradycardia.

μ-Opioid receptor in primary afferent nociceptors is identified to generate and persist opioid-induced tolerance, hyperalgesia and pronociceptive synaptic plasticity (Corder et al., 2017). Simultaneously, μ-opioid receptor antagonist at low-dose is sufficient to limit opioid-induced hyperalgesia without disrupting analgesia in rodent’s models of perioperative and chronic pain (Corder et al., 2017). Furthermore, intraoperative parallel administration of naloxone at ultra-low dose diminished remifentanil-related hyperalgesic response after thyroid surgery (Koo et al., 2017). As a fast-acting and long-acting μ-opioid receptor antagonist, nalmefene reaches its peak plasma concentration at 2–5 min after intravenous injection, and its plasma elimination half-life needs 11 h (Kim et al., 1997; Kyhl et al., 2016). Also, nalmefene exhibits a stronger antagonistic capacity for μ-opioid receptor than naloxone (Glass et al., 1994). Subsequently, nalmefene might be more appropriate for the management of clinical RIH. This is the first study in which preoperative nalmefene 0.20 μg kg^−1^ prevented RIH but not impaired postoperative pain development, as characterized by the increase of mechanical hyperalgesic threshold and the decrease of hyperalgesic area, without altering pain intensity and analgesics consumption after surgery. More strikingly, our present study, for the first time, identified that preoperative combination of dexmedetomidine 0.25 μg kg^−1^ and nalmefene 0.10 μg kg^−1^ successfully reduced hyperalgesia, postoperative pain, as well as the need for analgesics application after surgery with remifentanil infusion. Furthermore, patients treated with this combination experienced less intraoperative bradycardia and faster post-anesthesia recovery than those injected with dexmedetomidine 0.50 μg kg^−1^ alone, despite the similar efficacy of anti-hypernociception. The reduction in dexmedetomidine-related side-effects might be attributed to the combination of dexmedetomidine and nalmefene at ultra-low doses.

Although we previously demonstrated the prevention of RIH by the combination of butorphanol and flurbiprofen axetil, this therapy is inappropriate for patients with somnolence, dizziness, active peptic ulcer, recent gastrointestinal bleeding, renal dysfunction, pregnancy, or allergy to aspirin (Zhang et al., 2016). Thus, pre-operative administration of nalmefene and dexmedetomidine might be as an alternative solution for RIH patients with contraindication to butorphanol or non-steroidal anti-inflammatory drugs. Still, the underlying mechanisms associated with the prophylactic effect on RIH warrant further investigation. A possible limitation of this current study is the failure to explore the optimal combination of dosages. Moreover, further trials are needed to ascertain whether these positive results are generalizable to other abdominal surgical populations. Additionally, we only collected outcomes for intraoperative and acute postoperative periods, despite the strong link between RIH and chronic post-surgical pain (Fletcher and Martinez, 2014; Araldi et al., 2015; de Hoogd et al., 2016).

In conclusion, the current findings suggest that preoperative therapy of nalmefene or dexmedetomidine successfully prevented RIH after surgery. The combination of both at low dose produced similar anti-hyperalgesic efficacy and reduced drug-related side-effects. Consequently, the combination strategies may emerge as a promising therapeutic candidate for RIH and postoperative pain in clinics.

## Data Availability

The original contributions presented in the study are included in the article/supplementary material, further inquiries can be directed to the corresponding authors.

## Abbreviations

ANOVA, analysis of variance; ASA, American Society of Anesthesiologists; BIS, bispectral index; HR, heart rate; MAC, minimal alveolar concentration; MAP, mean arterial blood pressure; NMDA, N-methyl-d-aspartate; NRS, numerical rating scale; PACU, postanesthetic care unit; PCA, patient-controlled analgesia; SD, standard deviation; RIH, remifentanil-induced hyperalgesia.
